# “Be kind to yourself”: an interview with Sebastian Groh on being a transgender early career scientist during a global pandemic

**DOI:** 10.1038/s42003-021-02389-3

**Published:** 2021-07-22

## Abstract

Dr. Sebastian Groh is a postdoctoral researcher at University College London, UK, and co-founder of Trans In STEM. We asked them about their experiences as an early career palaeontologist, and the challenges faced by transgender scientists in academia.

**Figure:**
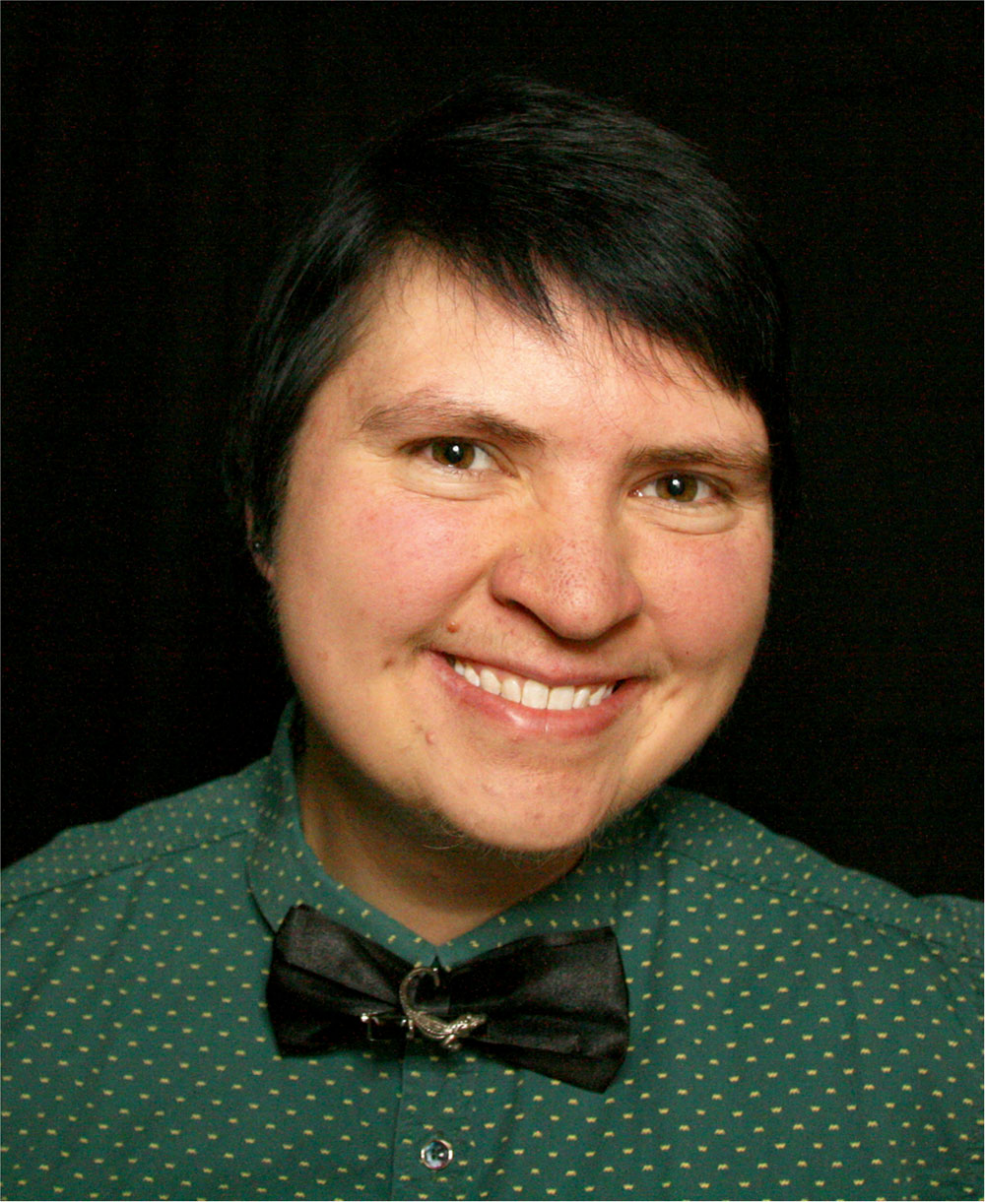
Sebastian Groh

1) Hi Seb, can you tell us about your research interests?

I have two major fields of work that I am interested in! The first is the evolution of crocodiles, which I also did my Ph.D in. My main group of interest is Neosuchia, which encompasses all modern members of the clade (alligators, crocodiles and gharials) and lots of extinct relatives. They evolved at roughly the same time as dinosaurs but formed their own group that constitutes a sister group to dinosaurs. Crocodiles & co. are incredibly fascinating, since they used to be far more diverse in the past, ranging from very small to gigantic sizes, with different habitat preferences (from terrestrial to marine) and different dietary requirements (including insectivorous and herbivorous crocs). I want to know how and when this diversity evolved and how they changed throughout earth’s history.

My second interest lies in the more general methodology that we use to reconstruct the evolutionary history of species and species groups. This includes phylogenetics (determining the relationships between species, including those already extinct), time-scaling (determining the timing of evolutionary events) and biogeography (determining the geographical locations where evolutionary events took place and how different species migrated across landmasses to achieve the distributions we see in the fossil record and today). I want to work on improving how we use these methods so we can get more accurate results from the data we have.

2) How has the global pandemic affected palaeontology as a whole, particularly for early career researchers?

As with most other areas, the pandemic has thrown the inequalities that exist in academia as a whole and in science into stark relief. Some people have experienced very little impact besides the general anxiety and associated struggles that obviously came with the pandemic, especially those whose work is already mostly computer and literature-based. Others experienced delays with fieldwork or collection-based work in museums, leading to the delay of a number of research projects. But as with all other fields, people already under pressure (such as those with caring responsibilities, for example) and/or from marginalised groups have been starkly impacted by the pandemic—this goes for Ph.D students and early career researchers who have experienced major delays due to not being able to see museum specimens or doing research, those with teaching responsibilities who have spent months upon months converting their courses and assessments into online teaching, leaving no time for research or other things. In addition, being from marginalised backgrounds and/or having other pressures often means that mental health has taken a stark dip during the pandemic, making it impossible to shoulder the usual workload whilst also coping with the immense mental pressure (and the isolation that stems from not being able to see your family and friends). Add to that the fact that almost all early career researchers are employed on precarious, short-term contracts, it’s almost a miracle that any of us got any work done at all.

I know many people (all from some sort of marginalised/minority backgrounds), myself included, who have become very disillusioned with academia and for whom the pandemic was probably the final straw. Almost all of us don’t think they will have a place in academia post-pandemic, unless things change radically.

3) Palaeontology has progressed a lot over the last decade with new method developments and more ambitious questions being asked of the fossil record. Where do you see the field going over the next few years?

I think the increased computational powers and the ability to deal with more and more data are going to have a major impact on palaeontology. CT scanning and using three-dimensional fossil data are already becoming really common in the discipline to answer evolutionary questions, and I think that trend is going to continue—perhaps, one day, we will simply be able to scan every single taxon we want to research and then use them holistically, for example, for building phylogenies, instead of having to do it all by hand (although there will always be the need for some quality control and someone with actual anatomical and taxonomical knowledge overseeing everything).

I also hope that we will continue to be more interdisciplinary—there are a lot of exciting questions that can be asked and answered once we start collaborating even more strongly than is already happening, with life sciences for example. There is a lot that we can learn from palaeontology that will help us with current conservation efforts, and there are major overlaps with other disciplines (for example the algorithms we use to build phylogenetic trees are very similar to those used in machine learning and artificial intelligence computing).

4) Can you tell us about your experiences as a transgender scientist?

If I had to summarise it in one word, it would be: exhausting. What all of us trans people in STEM actually want to do is to be able to concentrate on our science and to do the work that excites and delights us, but unfortunately this isn’t really possible, at least not without caveats.

From a work perspective, there are fieldwork sites we will never be able to visit, conferences we won’t be able to travel to, collaborations that will never happen because of something that is utterly outside of our control. At every meeting, every conference, we have to decide again and again – will we come out to these people (and oftentimes this is a decision between the safety of staying in the closet vs the extremely negative impact on mental health of staying in the closet)? Is it safe to come out? Are there gender-neutral toilets we could use? And so on. And even then, it is common to get misgendered or deadnamed, not to talk about the difficulty of changing your deadname on publications from before you came out.

For those who decide to transition, these factors are compounded by other things—for one, transitioning and things like name & gender changes, as well as surgeries and the like are very expensive and at least in the UK, the waitlists on the public health system are now so long (4+ years or more to even be seen for the first time) that going private is the only option. I’m estimating that by the end of my current postdoctoral contract I’ll have spent a little less than £10,000 on being trans (and I am very lucky to be privileged and have the support of my family to offset a lot of these costs, otherwise I couldn’t afford it. I certainly don’t have savings anywhere near that sum!).

Secondly, being trans and transitioning (although keep in mind—not every trans person can or will want to transition and not transitioning doesn’t make people any less trans!) can be mentally and physically taxing which comes on top of the career insecurity one faces as an early career researcher, and the challenges that come with being an autistic person in my case. You are trying to cope with these changes—a puberty on speedrun (with a few more physically painful side effects if you are unlucky) and a global pandemic, the toxicity of academia and precarity of being an early career researcher—whilst also trying to establish some sort of research career and delivering satisfactory teaching to your undergraduates. And all of this under the constant onslaught of negative media pieces about trans people, as well as academics at our own workplaces that write think pieces treating us like an abstract idea rather than the real people we are and insist we shouldn’t be allowed to exist.

Having said that, it is important to acknowledge that I can still write this from a relatively privileged position—whilst fixed-term, my current work contract does last beyond the pandemic. I have supportive parents and am white and transmasc—a lot of the vitriol in the media is aimed particularly at trans women, not to speak about the immense mental pressure that racism and other factors can add to this mix.

This all sounds rather bleak and depressing but there are, of course, positives to go with it, too—I am still, on the whole, far happier now at 30 than I have been for most of the first three decades of my life. I have had the privilege of meeting some absolutely amazing people through EDI initiatives and online spaces and seen first hand the generosity and beauty in this community and the willingness to help that is there both in allies and fellow trans people and scientists. There is a lot of good besides all the negative and it is what keeps me going.

5) Historically, the field has a poor record for diversity. Can you highlight some key initiatives working to correct this discrepancy?

There are quite a few really great initiatives out there, so apologies in advance if I forget any! Pride in STEM, the STEM Village and 500 Queer Scientists have been doing some stellar work to bring more queer people into the spotlight in the sciences and highlight all the issues of what should still be done. I also co-founded Trans in STEM which aims to highlight trans people in STEM and both the amazing work we do and the challenges we face.

Tiger in STEMM do fantastic work to advance diversity in STEM in particular across the UK, penning letters and publishing papers and press releases to help with those goals. 500 Women Scientists works to connect women in STEM all around the globe and improve the academic environment for them. BlackAFinSTEM works to promote visibility of black people in STEM in particular and showcases some amazing scientists. DisabledinSTEM provides a safe space for disabled people in STEM.

There are many more—Twitter and hashtags are a great way to find them!

6) What advice would you give to young scientists struggling with their identity?

Find kindred spirits! Networks (be they digital or in person) can be real lifesavers. There is a wealth of Discord servers, online forums, and more out there that you can join. Once you start talking to others you realise that a lot of the problems you experience are shared by others, and it’s always easier to commiserate and find solutions together! Community is incredibly important.

Secondly—be kind to yourself! Figuring out your identity is a complicated process that never really stops (take it from a guy in his thirties who figured out they might be trans with 26 and still isn’t sure about their exact gender or sexuality!). Take all the time you need—experiment around, explore, find whatever is most comfortable for you. We’re here and waiting for you with open arms whenever you feel safe and comfortable enough to come out. It’s a scary thing and it never really stops, but there are also a lot of exciting things and lots of people who will love and support you just the way you are.

*This interview was conducted by Associate Editor Luke R. Grinham*.

